# Computation in Dynamically Bounded Asymmetric Systems

**DOI:** 10.1371/journal.pcbi.1004039

**Published:** 2015-01-24

**Authors:** Ueli Rutishauser, Jean-Jacques Slotine, Rodney Douglas

**Affiliations:** 1 Computation and Neural Systems, California Institute of Technology, Pasadena, California, United States of America; 2 Division of Biology and Biological Engineering, California Institute of Technology, Pasadena, California, United States of America; 3 Departments of Neurosurgery, Neurology and Biomedical Sciences, Cedars-Sinai Medical Center, Los Angeles, California, United States of America; 4 Nonlinear Systems Laboratory, Department of Mechanical Engineering, Massachusetts Institute of Technology, Cambridge, Massachusetts, United States of America; 5 Institute of Neuroinformatics, University and ETH Zurich, Zurich, Switzerland; Indiana University, UNITED STATES

## Abstract

Previous explanations of computations performed by recurrent networks have focused on symmetrically connected saturating neurons and their convergence toward attractors. Here we analyze the behavior of asymmetrical connected networks of linear threshold neurons, whose positive response is unbounded. We show that, for a wide range of parameters, this asymmetry brings interesting and computationally useful dynamical properties. When driven by input, the network explores potential solutions through highly unstable ‘expansion’ dynamics. This expansion is steered and constrained by negative divergence of the dynamics, which ensures that the dimensionality of the solution space continues to reduce until an acceptable solution manifold is reached. Then the system contracts stably on this manifold towards its final solution trajectory. The unstable positive feedback and cross inhibition that underlie expansion and divergence are common motifs in molecular and neuronal networks. Therefore we propose that very simple organizational constraints that combine these motifs can lead to spontaneous computation and so to the spontaneous modification of entropy that is characteristic of living systems.

## Author Summary

Biological systems are obviously able to process abstract information on the states of neuronal and molecular networks. However, the concepts and principles of such biological computation are poorly understood by comparison with technological computing. A key concept in models of biological computation has been the attractor of dynamical systems, and much progress has been made in describing the conditions under which attractors exist, and their stability. Instead, we show here for a broad class of asymmetrically connected networks that it is the unstable dynamics of the system that drive its computation, and we develop new analytical tools to describe the kinds of unstable dynamics that support this computation in our model. In particular we explore the conditions under which networks will exhibit unstable expansion of their dynamics, and how these can be steered and constrained so that the trajectory implements a specific computation. Importantly, the underlying computational elements of the network are not themselves stable. Instead, the overall boundedness of the system is provided by the asymmetrical coupling between excitatory and inhibitory elements commonly observed in neuronal and molecular networks. This inherent boundedness permits the network to operate with the unstably high gain necessary to continually switch its states as it searches for a solution. We propose that very simple organizational constraints can lead to spontaneous computation, and thereby to the spontaneous modification of entropy that is characteristic of living systems.

## Introduction

The principles of biological computation are not well understood. Although the Turing Machine and related concepts [1–3] have provided powerful models for understanding and developing technological computing, they have provided less insight for biological computation because they generally assume that the machines themselves, as well as their initial program and data are granted as input. In contrast, the organization of states and transitions of the biological process arise out of phylogenetic and ontogenetic configuration processes and execute autonomously without the intervention of an intelligent external programmer and controller being necessary to supply already encoded organizationally relevant information. Our goal here is to make steps towards understanding biological computation [4–6], by considering the behavior of a simple non-linear dynamical system composed of asymmetrically inter-connected linear-threshold neurons. We suppose that such computations entail a mapping from some input towards a limited (low entropy) region of phase space, which is the solution [7]. We do not suppose that the computational goal is known—only that computation must conform to this basic entropy reducing process. Here we describe the organizational constraints that make such spontaneous computation possible.

Previous authors have explained neural network computation in terms of the convergence of special dynamical systems, and emphasized the attractors to which they converge [8–13]. For example, Hopfield [9, 10] has shown how and why the dynamics of symmetrically connected neurons with saturating outputs converge to attractor states; and others have offered similar insights for symmetrically connected linear threshold neurons [14–16]. However, interactions between inhibitory and excitatory neurons are clearly asymmetric, making these studies ill suited to study biological computation. To the extent that asymmetrical networks have been considered at all, this has been through assumptions that reduce asymmetrical networks to approximate symmetry. By contrast, we consider here the dynamics of fully asymmetrical networks, and discover that asymmetry contributes strongly to computational behavior.

The scope of our work is restricted to recurrent neural networks with asymmetric coupling that express an important and ubiquitous behavior: soft winner-take-all (sWTA) dynamics. We present a formal account of the response of these networks to exogenous perturbations and use a form of non-linear stability analysis (contraction analysis [17]) to characterize the itinerant transients than ensue, and which have useful interpretations in terms of neuronal computation and information theory. Contraction Theory offers a more flexible framework than the conventional Lyapunov approach to non-linear stability (See Methods for details). This is particularly the case for non-autonomous systems such as our network, in which external inputs can vary with time.

We explore particularly the behavior of network computation during the non-equilibrium phase, when the network is traversing its state-space seeking for a solution. We show that the ability of the network to explore potential solutions depends on highly unstable ’expansion’ dynamics driven by recurrent excitation. This expansion is steered and constrained by negative divergence of the dynamics, which ensures that the dimensionality of the solution space continues to reduce until an acceptable solution manifold is reached. The system then ’contracts’ stably on this manifold [17] towards its final solution trajectory, which is not necessarily converging to a fixed point. We argue that the simple principle of unstable expansion constrained by negative divergence provides the central organizing drive for more general autonomous biological systems from molecular networks, through neurons, to society.

Consider a simple network of non-linear neuron-like elements whose task it is to compute the solution to some problem. The states of the computation are encoded in the activations (firing rates) of the neurons, and the computational transitions between these states arise out of their synaptic interactions. The overall trajectory resulting from the successive transitions through its state space express its computation [18–20]. In current technological systems the hardware states of the system are encoded on binary nodes whose discrete states are imposed by signal restoring [21] circuitry [19]. This signal restoration is achieved by extremely high gain, so that a small input bias will drive the node into saturation at one of its two voltage limits. Biology rarely commands such sharply demarcated states and transitions. Instead, molecular and electrophysiological activation functions are often approximately sigmoidal (eg Hill functions, voltage dependent conductance, neuronal current-discharge curves, etc). However, neuronal systems do not typically run in saturation. The typical activation of a neuron is thresholded below, and above this threshold it makes use of only the lower part of its dynamic range. It very rarely enters saturation at the upper end of its activation range. Therefore, a suitable model for neuronal activation is a thresholded linear one. That is, their activity is bounded from below, but their positive activity is essentially unbounded (over any practical range of discharge). This is a very well studied model [14–16, 22].

**Figure 1:**
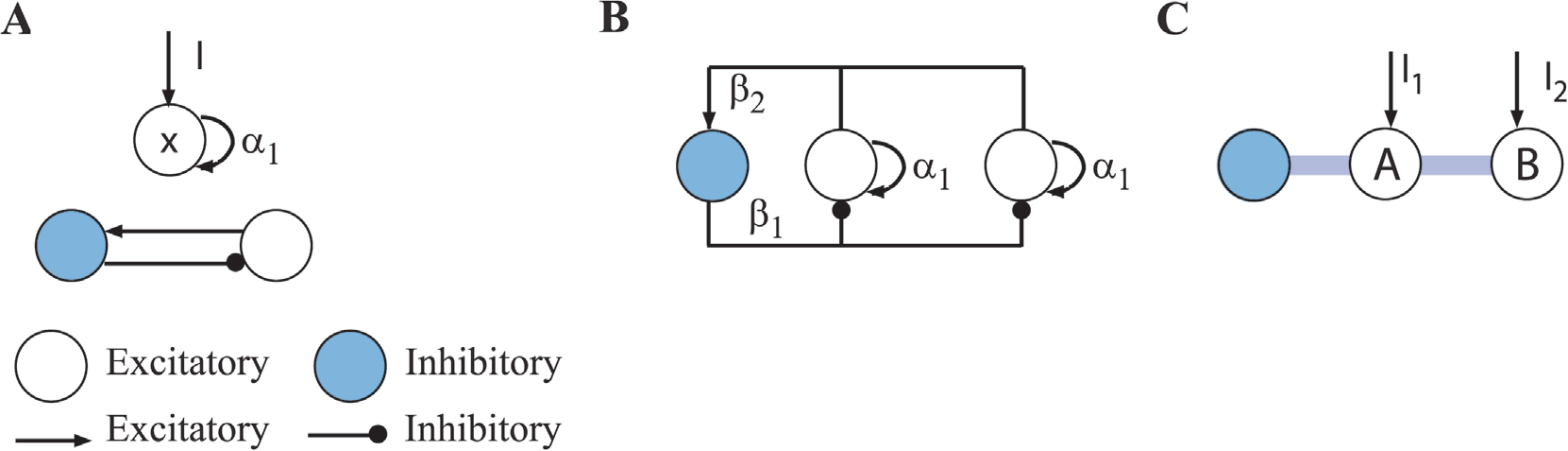
Circuit motifs and simple circuit composed of motifs. (A) Circuit motifs are excitatory self-recurrence (top) and shared inhibition (bottom). *I_i_* denotes an external input. (B) Connectivity of a simple WTA circuit, consisting of two excitatory units that compete through shared inhibition. (C) More compact notation to denote the circuit shown in (B). Each excitatory element receives an external input.

As in our previous work, the neuronal network model is composed of thresholded linear neuron-like units coupled through positive (excitatory) and negative (inhibitory) connections (see Fig. 1a). The unbounded positive range of neuron activation implies that the global stability of networks of these neurons must arise out of their collective interactions rather than from saturation of their individual activation functions as assumed by for example [9, 10]. The key interaction here is the inhibitory feedback, which must at least ensure that not all neurons can simultaneously increase their activation [16]. Previous studies of such models have focused on the mathematically more tractable case in which the connections between neurons are symmetrical, and have no transmission delays. Our networks, by contrast, need not be symmetrical and may have transmission delays. Indeed, the asymmetry of connections will be used to computational advantage, not offered by symmetrical networks.

## Results

### Network model

The network contains two fundamental circuit motifs (Fig. 1A): excitatory neurons that project both onto themselves and others, and inhibitory neurons which receive excitatory input from the same neurons that they inhibit. Several instances of these motifs together compose the general recurrent circuit that we investigate. In its simplest form, this recurrent network has the following form. There are N neurons, N-1 of which are excitatory and one (index N) is inhibitory (Fig. 1B,C). The excitatory neurons *x*_*i* ≠ *N*_ receive an optional external input *I*_*i*_, and excitatory feedback *α* from themself and nearby excitatory neurons (*α*_1_ and *α*_2_, respectively). The single inhibitory neuron *x*_*n*_ sums the *β*_2_ weighted input from all the excitatory neurons, and sends a common *β*_1_ inhibitory signal to each of its excitatory neurons. Each neuron has a resistive constant leak term *G*_*i*_.

The dynamics of this simple network are:

$$\tau {\dot{x}}_{i}+G_{i}x_{i}=f\left(I_{i}\left(t\right)+\alpha_{1}x_{i}+\alpha_{2}x_{i-1}+\alpha_{2}x_{i+1}-\beta_{1}x_{N}\right)$$ (1)

$$\tau {\dot{x}}_{N}+G_{i}x_{N}=f\left(\beta_{2}\sum_{j=1}^{N-1}x_{j}\right)$$ (2)

where *f*(*x*) is a non-saturating rectification non-linearity. Here, we take *f*(*x*) = *max*(*x*, 0), making our neurons linear threshold neurons (LTNs).

As long as the key parameters (*α*_*i*_, *β*_*i*_) satisfy rather broad constraints [23], this network behaves as a soft winner-take-all by allowing only a small number of active neurons to emerge, and that solution depends on the particular input pattern *I*(*t*). After convergence to the solution, the active neurons (winners) will express an amplified version of the input *I*(*t*), that is they remain sensitive to the input even if it changes.

After convergence to steady state in response to constant input *I*_*i*_ > 0, the activation of the winner *i* is:

$$x_{i}=\frac{I_{i}}{1-\alpha_{1}+\beta_{1}\beta_{2}}=gI_{i}$$ (3)

where $g=\frac{1}{1-\alpha_{1}+\beta_{1}\beta_{2}}$ is the gain of the network. Importantly, this gain can be *g* > > 1 due to excitatory recurrence, which amplifies the signal in a manner controlled by the feedback loop. While above assumes *α*_2_ = 0, similar arguments hold without this assumption [23].

The dynamics of the non-linear system are $\tau \dot{x}=f(\mathrm{Wx}+I(t))-\mathrm{Gx}$ (where **f** applies the scalar function *f*(*x*) component-wise). For example, a minimal WTA with two excitatory and one inhibitory units, **x** = [*x*_1_, *x*_2_, *x*_3_] (Fig. 1B,C), the weight matrix **W** is

$$W=\left[\begin{array}{ccc} \alpha_{1} & 0 & -\;\beta_{1}\\ 0 & \alpha_{1} & -\;\beta_{1}\\ \beta_{2} & \beta_{2} & 0 \end{array}\right]$$ (4)

where **G** = diag(*G*_1_, …, *G_n_*) is a diagonal matrix containing the dissipative leak terms for each unit. The time constant of the system is $\frac{\tau }{G}$. Now we consider the conditions under which the non-linear system is guaranteed to be stable but at the same time powerful, i.e. permitting high gain.

### Instability drives computation

Contraction Theory assesses the stability of non-linear systems $\dot{x}=f(x,t)$ using virtual displacements of its state at any point with respect to a chosen uniformly positive definite metric *M*(*x*, *t*), obtained by a transformation Θ(*x*, *t*) (see Methods). The key Theorem 2 of [17] asserts that if the temporal evolution of *any* virtual displacement in this metric space tends to zero, then *all* other displacement trajectories in this space will also contract (shrink) to the same (common) trajectory.

For our present case, this theorem implies (see Methods) that the trajectories of a system of neurons with Jacobian **J** are exponentially contracting if

$$\Theta J\Theta^{-1}<0$$ (5)

The Jacobian **J** has dimension *N* and describes the connection matrix of the network and **Θ** is a transformation matrix that provides a suitable choice of coordinates.

For a WTA with weight matrix **W**, the Jacobian is

$$J=\frac{\partial f}{\partial x}=\Sigma W-G$$ (6)

where Σ = diag(*σ*_1_, …, *σ*_*n*_) is a switching matrix, whose diagonal elements *σ*_*i*_ ∈ 0, 1 indicate whether unit *i* is currently active or not.

Following [16] we will call the subset of N neurons that are currently above threshold the active set. Those that are inactive cannot contribute to the dynamics of the network. Thus, we may distinguish between the static anatomical connectivity *W* of the network, and the dynamic functional connectivity that involves only the subset of currently active neurons. This active set is described by the switch matrix Σ. The Jacobian expresses which units contribute to the dynamics at any point of time, and is thus a reflection of functional rather than anatomical connectivity. Each possible effective weight matrix has a corresponding effective Jacobian. Thus, Σ*W* − **G** is the effective (functional) Jacobian of the network, in which only active units have an influence on the dynamics.

The active sets may be either stable or unstable. In previous work on the hybrid analog and digital nature of processing by symmetric networks, Hahnloser et al. refer to such sets as active and forbidden, respectively [16]. They further show that the eigenvectors associated with positive eigenvalues of forbidden sets are mixed. That is, the eigenvector contains at least one component whose sign is opposite to the remainder of the components. This property ensures that a neuron will finally fall beneath threshold, and that the composition of the active set must change. We now generalize this concept to our asymmetrical networks, and refer to permitted and forbidden subspaces rather than sets, to emphasize the changes in the space in which the computational dynamics play out.

It is the instability (exponential divergence of neighboring trajectories) of the forbidden subspaces rather than stability that drives the computational process. This instability can be approached also through Theorem 2 of [17], which notes that if the minimal eigenvalue of the symmetric part of the Jacobian is strictly positive, then it follows that two neighboring trajectories will diverge exponentially. We will use ’expanding’ to refer to this unstable, exponentially diverging behavior of a set of neurons to avoid confusion with Gaussian divergence, which we will need to invoke in a different context, below.

Thus, we will say that the dynamics of a set of active neurons is expanding if

$$\Theta VJV^{T}\Theta^{-1}>0$$ (7)

where **V** is a projection matrix which describes subspaces that are unstable. For example, for a circuit with two excitatory units that cannot both be simultaneously active, **V** is

$$V=\left[\begin{array}{ccc} \alpha & 0 & -\;\beta_{1}\\ 0 & \alpha & -\;\beta_{1} \end{array}\right]$$ (8)

and **Θ** is a metric. The constraint (7) asserts that the system escapes the unstable subspaces where **Vx** is constant. This guarantees that For **V** as defined above $\text{Vx}=\left[\begin{array}{c} \alpha x_{1}-\beta_{1}x_{3}\\ \alpha x_{2}-\beta_{1}x_{3} \end{array}\right]$. Each row represents one excitatory unit. Guaranteeing that **Vx** cannot remain constant for a particular subset implements the requirement that for a subspace to be forbidden, it cannot be a steady state because if it were **Vx** would remain constant after convergence.

The parameter conditions under which Eqn 7 holds, are given in detail in [24]. Importantly, these conditions guarantee that when the dynamics of our asymmetric network occupies an unstable subspace, all eigenvectors are mixed (see Methods for proof). Consequently, as in the symmetric case of [16, 25], this unstable subspace will be left (it is forbidden ), because one unit will fall beneath its threshold exponentially quickly and so become inactive.

### Divergence quenches computational instability

**Figure 2:**
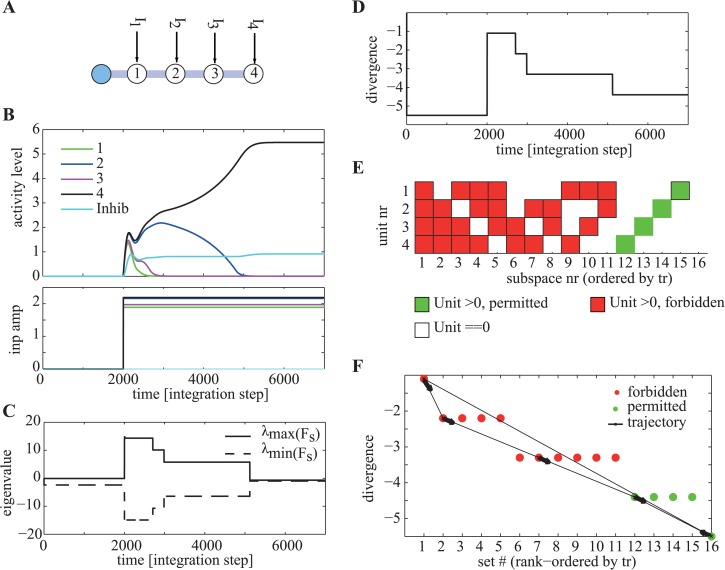
Illustration of key concepts to describe biological computation. (A) Connectivity of the circuit used in this figure. (B–F) Simulation results, with parameters *α*1 = 1.2, *β*_1_ = 3, *β*_2_ = 0.25, *G*_5_ = 1.5, *G*_1..4_ = 1.1. (B) Activity levels (top) as a function of time for the units shown in (A) and external inputs provided (bottom). After stimulus onset at *t* = 2000, the network selectively amplifies the unit with the maximal input while suppressing all others. (C) Maximal and minimal eigenvalue of the functional connectivity **F**_*S*_ active at every point of time. At *t* = 5000 the system enters a permitted subspace, as indicated by the max eigenvalue becoming negative. (D) Divergence as a function of time. The divergence decreases with every subsapce transition. (E) Illustration of all possible subspaces (sets) of the network. Subspaces are ordered by their divergence. For these parameters, only subspaces with at most one unit above threshold are permitted (green) whereas the others are forbidden (red). (F) Divergence as a function of subspace number (red and green circles) as well as trajectory through set space for the simulation illustrated in (B–D). Note how the divergence decreases with each transition, except when the input changes (set 16 is the off state).

The dynamics of our asymmetric networks can now be explained in terms of the contraction theory framework outlined above. Consider a simple network consisting of *N* = 5 neurons, one of which is inhibitory and enforces competition through shared inhibition (Fig. 2A). For suitable parameters that satisfy the contraction constraints (see [24]), this network will contract towards a steady state for any input. The steady state will be such that the network amplifies one of the inputs while suppressing all others (for example, Fig. 2B). During this process of output selection and amplification, the network passes through a sequence of transformations, at each of which a different subset of units becomes active whereas the remainder are driven beneath threshold and so are inactive. These transformations continue while the network is in its expansion phase and cease when the network contracts. The network is in the expansion phase while the effective Jacobian is positive definite, and contracts when the Jacobian becomes negative definite (Fig. 2C).

The computational process of selecting a solution, conditional on inputs and synaptic constraints, involves testing successive subspaces, expressed as changing patterns of neuronal activation (Fig. 2B). Subspaces do not offer a solution (are forbidden) if for that subspace **VJV** ^*T*^ is positive definite. In this case its dynamics are expanding, and because all eigenvectors in this subspace are mixed, the subspace will finally switch. Subspaces that offer solutions (are permitted) will have an effective Jacobian that is negative definite in some metric (they are contracting), and so the system will not leave such a subspace provided that the input remains fixed. Note that there can be subspaces whose Jacobian is neither positive nor negative definite, which are then neither permitted or forbidden. However, by definition, the networks we describe assure that each possible subspace is either permitted or forbidden.

Consider the case in which the computational process begins in a forbidden subspace (Fig. 2F). The process then passes successively through several forbidden subspaces before reaching a permitted subspace. Two properties ensure this remarkable orderly progression towards a permitted subspace. Firstly, the process is driven by the instability that ensures that forbidden subspaces are left. However the trajectory is associated with a progressive reduction in the maximum positive eigenvalue of the active set (Fig. 2C).

Secondly, an orderly progression through forbidden subspaces is ensured by systematic reduction of the state space through Gaussian divergence. Gauss’ theorem

$$\frac{d}{dt}\delta V=\mathrm{div}\left(\frac{d}{\mathrm{dt}}\delta z\right)\;\delta V$$ (9)

asserts that, in the absence of random fluctuations, any volume element δ*V* shrinks exponentially to zero for uniformly negative definite $\text{div}(\frac{\text{d}}{\text{dt}}\delta \text{z})$. This implies convergence to an (*n*−1) dimensional manifold rather than to a single trajectory. For our system, the Gaussian divergence is the trace of the Jacobian **J** or equivalently the sum of all of its eigenvalues. Note that this is a much weaker requirement than full contraction. In particular, we are concerned about the trace of the effective Jacobian, which is the Jacobian of only the active elements, because the inactive elements (those below threshold) do not contribute to the dynamics.

The divergence quantifies the rate at which the volume shrinks (exponential) in its *n* dimensional manifold towards a (*n*−1) dimensional manifold. The system will enter the (*n*−1) dimensional and continue its evolution in this new active subset, and so on, until the reduced dimensional system finally enters a permitted subspace (which is contracting). Note that here we refer to the dimensionality of the system dynamics. This dimensionality is not necessarily equal to the number of active units (i.e. at steady state or when one of the units is constant).

Note that negative Gaussian divergence does not follow automatically from expansion. In fact most expanding sets will not have negative Gaussian divergence, and so will not be forbidden

For example consider the linear system $\dot{\text{x}}=\text{Wx}$ with $W=\left[\begin{array}{ccc} 1 & 0 & 0\\ 0 & 1 & 0\\ 0 & 0 & 1 \end{array}\right]$. This system is expanding but it does not have negative divergence.

For orderly computation to proceed, such sets must not exist. We require that all forbidden subspaces are expanding as defined by contraction theory as well as have negative Gaussian divergence. Indeed, for our LTN networks all forbidden subspaces are guaranteed to satisfy both these properties.

### Computation is steered by the rotational dynamics induced by asymmetric connections

Because their positive output is unbounded, linear thresholded neurons are essentially insensitive to the range of their positive inputs. Networks of these neurons amplify their inputs in a scale-free manner, according to the slope of their activation functions and the network gain induced by their connections. As explained above, this high gain drives the exploration and selection dynamics of the computational process. The network harnesses its high gain to steer the computation through its asymmetric connections. Indeed, the asymmetric nature of the connectivity in our network is central to its operation, and not a minor deviation from symmetry (approximate symmetry, [26]).

The interplay between high-gain and steering can be appreciated by considering the behavior of the system within one of the subspaces of its computation.

Consider a linear system of the form $\dot{\text{x}}=\text{f}(\text{x},t)$, which can be written as $\dot{\text{x}}=\text{M}x+\text{u}$ where **M** is a matrix of connection weights and **u** a vector of constant inputs. For the example of a WTA with 2 excitatory units and 1 inhibitory unit,

$$M=\left[\begin{array}{ccc} l_{1}\alpha -G & 0 & -\;l_{1}\beta_{1}\\ 0 & l_{2}\alpha -G & -\;l_{2}\beta_{1}\\ l_{3}\beta_{2} & l_{3}\beta_{2} & -G \end{array}\right]$$ (10)

Setting **M** = **M**_1_+**M**_2_ and defining

$$M_{1}=\left[\begin{array}{ccc} l_{1}\alpha -G & 0 & 0\\ 0 & l_{2}\alpha -G & 0\\ 0 & 0 & -G \end{array}\right]$$ (11)

$$M_{2}=\left[\begin{array}{ccc} 0 & 0 & -\;l_{1}\beta_{1}\\ 0 & 0 & -\;l_{2}\beta_{1}\\ l_{3}\beta_{2} & l_{3}\beta_{2} & 0 \end{array}\right]$$ (12)

provides a decomposition into a component with negative divergence and zero divergence (see methods for an example).

Any vector field *f*(**x**) can be written as the sum of a gradient field ∇*V*(*x*) and a rotational vector field *ρ*(*x*), i.e. a vector field whose divergence is zero. In analogy, $f(\text{x})=\dot{\text{x}}={\text{M}}_{1}x+\text{u}+{\text{M}}_{2}x$ where ∇*V*(*x*) = **M**_1_ *x*+**u** and *ρ*(*x*) = **M**_2_ *x*.

For our network **M**_1_ is the expansion/contraction component and **M**_2_ is the rotational component. This is an application of the Helmholtz decomposition theorem to our network [27, 28]. These two matrices relate to two functional components in the network architecture: The excitatory recurrence plus input, and the inhibitory recurrence. The first component provides the negative divergence that defines the gradient of computation, while the second steers its direction as follows.

Since **M**_2_ has a divergence of zero, this component is rotational. If, in addition, **M**_2_ is skew-symmetric so that $-{\text{M}}_{2}={\text{M}}_{2}^{T}$, the system is rotating, and the eigenvalues of **M**_2_ will be imaginary only. In general, *β*_2_ ≠ −*β*_1_ and **M**_2_ is thus not skew-symmetric. However, note that a transform of the form **Φf**(**x**)**Φ** ^−1^ can be found that makes **M**_2_ skew-symmetric. Because such transform does not change the eigenvalues of **M**_1_, it will not change its divergence either. For above example,

$$\Phi =\left[\begin{array}{ccc} 1 & 0 & 0\\ 0 & 1 & 0\\ 0 & 0 & \sqrt{\frac{\beta_{1}}{\beta_{2}}} \end{array}\right]$$ (13)

which will result in a version of **M**_2_ that is skew-symmetric

$$\Phi M_{2}\Phi^{-1}=\left[\begin{array}{ccc} 0 & 0 & -\sqrt{\beta_{1}\beta_{2}}\\ 0 & 0 & -\sqrt{\beta_{1}\beta_{2}}\\ \sqrt{\beta_{1}\beta_{2}} & \sqrt{\beta_{1}\beta_{2}} & 0 \end{array}\right]$$ (14)

The same transformation to a different metric has to be applied to **M**_1_ as well, but as both **M**_1_ and **Φ** are diagonal this will leave **M**_1_ unchanged, **M**_1_ = **ΦM**_1_ **Φ** ^−1^.

The orderly progression through the forbidden subspaces can be understood in this framework: The negative divergence provided by **M**_1_ enforces an exponential reduction of any volume of the state space while the rotational component **M**_2_ enforces exploration of the state space by directing (steering) the dynamics.

The stability of the permitted subspaces can also be understood in this framework. Permitted subspaces are contracting, despite the strong self-recurrence of the excitatory elements that results in positive on-diagonal elements. This high gain, which is necessary for computation, is kept under control by a fast enough rotational component **M**_2_ which ensures stability. This can be seen directly by considering one of the constraints imposed by contraction analysis on valid parameters affecting an individual neuron: keeping $\alpha <2\sqrt{\beta_{1}\beta_{2}}$ guarantees that the system rotates sufficiently fast to remain stable.

Note that both **M**_1_ and **M**_2_ change dynamically as a function of the currently active subspace. Thus, the direction and strength of the divergence and rotation change continuously as a function of both the currently active set as well as the input.

### Changes of entropy during computation

While the network is in the expansion phase, the volume of the state space in which the dynamics of the network evolves is shrinking. This process depends on initial conditions and external inputs. Consequently, the sequence by which dimensions are removed from state space is sensitive to initial conditions. To quantify the behavior of a network for arbitrary initial conditions it is useful to compute its information entropy *H*(*t*) as a function of time (see methods). The smaller *H*(*t*), the smaller the uncertainty about which subspace the network occupies at time *t*. Once *H*(*t*) ceases to change, the network has converged. To reduce state uncertainty, and so to reduce entropy, is fundamentally what it means to compute [7].

**Figure 3:**
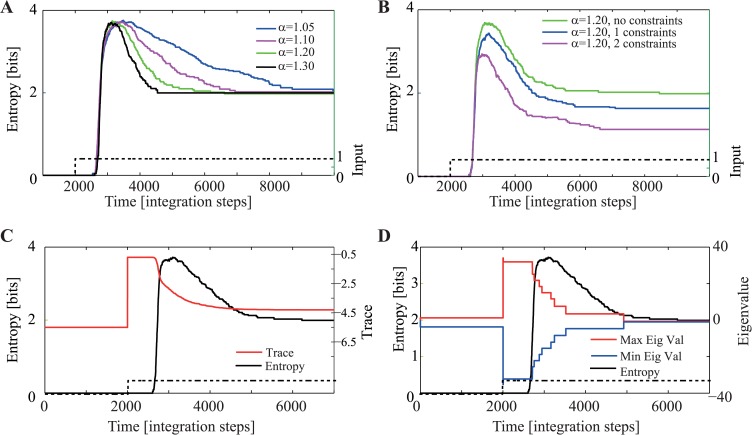
Spontaneous increase followed by reduction of state entropy during the expansion phase. Random inputs were provided to the 5-node network as shown in Fig. 2. Each input *I_i_* was chosen i.i.d from a normal distribution with *μ* = 6 and *σ* = 0.25. (A) Entropy as a function of time and gain of the network. Higher gains are associated with faster increases and reductions of entropy but converge to the same asymptotic entropy. This indicates that each permitted subspace is reached with equal probability. (B) Adding constraints reduces the peak and asymptotic entropy. The more constraints that are added, the larger is the reduction in entropy. (C) Comparison of time-course of average entropy and divergence (trace). (D) Comparison of time-course of average entropy and eigenvalues (min, max). Notice how both the divergence (C) and the eigenvalues (D) reach their maximal values well before the entropy reaches its maximum.

The entropy remains 0 immediately after the application of external inputs to the network, because the same “all on” subspace is reached regardless of initial conditions. Thereafter the network begins to transition through the hierarchy of forbidden subspaces (Fig. 2F). This process initially increases the entropy as the network explores different subspaces. Eventually, after attaining peak entropy, the network reduces entropy as it converges towards one of its few permitted subspaces. Fig. 3 illustrates this process for the network shown in Fig. 2A.

Increasing the gain by increasing the value of the self-recurrence *α* increases the speed by which entropy is changed but not its asymptotic value. This means that all permitted subspaces are equally likely to be the solution but that solutions are found mode quickly with higher gain. Adding additional constraints through excitatory connections makes some permitted subspaces more likely to be the solution, and so the asymptotic entropy is lower (see Fig. 3B, where a connection *α*_2_ = 0.2 from unit 1 to 2 and *α*_3_ = 0.2 from unit 4 to 2 was added).

Note how the number of constraints and the gain of the network are systematically related to both the increasing and decreasing components of *H*(*t*). For example, increasing the gain leads to a more rapid increase of entropy, reaching peak earlier and decaying faster towards the asymptotic value (Fig. 3A). Also, adding constraints results in smaller peak entropy, indicating that the additional constraints limited the overall complexity of the computation throughout (Fig. 3B).

The network reaches maximal entropy when there exists the largest number of forbidden subspaces having the same divergence. This occurs always for an intermediate value of divergence, because then there occurs the largest number of subspaces having an equal number of units active and inactive. This can be seen by considering $\underset{k}{\text{arg max}}\;\left(\begin{array}{c} N\\ k \end{array}\right)=\frac{N}{2}$ (if N is even), i.e. the network will have maximal entropy when the number of active units is 50%. Numerically, this can be seen by comparing the time-course of the maximal positive eigenvalue or the divergence with that of the time-course of the entropy (Fig. 3C,D).

Overall, *H*(*t*) demonstrates the dynamics of the computational process, which begins in the same state (at its extreme, all units on), and then proceeds to explore forbidden subspaces in a systematic fashion by first expanding and than contracting towards a permitted subspace.

### Structure and steering of computation

We will now use the concepts introduced in the preceding sections to explain how our circuits compute and how this understanding can be utilized to systematically alter the computation through external inputs and wiring changes in the network.

**Figure 4:**
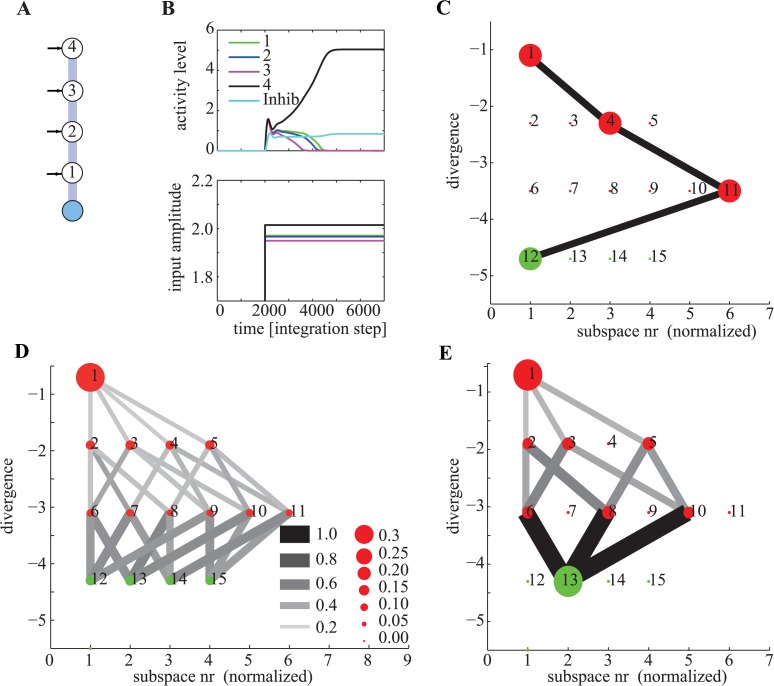
Hierarchy of subspaces and steering of computation. (A) 5-node network, using notation from Fig. 2. (B) Simulation of an individual run. Top shows the state and bottom the inputs to the network. The unit with the maximal input wins (unit 4). (C) Trajectory through state space for the simulation shown in (B). Each subspace is numbered and plotted as a function of its divergence. Red and green dots indicate forbidden and permitted subspaces respectively. Numbers inside the dots are the subspace (set) numbers (see Fig. 2E). (C–D) Transition probabilities for the same network, simulated with 1000 different random inputs. Connected subspaces are subsets between which the network can transition, in the direction that reduces divergence (gray lines). The size of dots and lines indicates the likelihood that a subset will be visited or a transition executed, respectively. The subset with most negative divergence is the zero set (all units off), which is not shown. (C) All transitions made by the network. Depending on the value of the input, the network will reach one of the permitted subspaces. Notice the strict hierarchy: all transitions were towards subspaces with lower divergence and only a subset of all possible transitions are possible. (D) All transitions the network made, conditional on that subspace 13 was the solution. Note that this subspace cannot be reached from some subspaces (such as nr 7).

Provided that the external input remains constant, the network proceeds in an orderly and directed fashion through a sequence of forbidden subspaces. This sequence of steps is guaranteed to not revisit subspaces already explored, because when in a forbidden subspace *S*_1_ with divergence *d*_1_, the next subspace *S*_2_ that the network enters must have more negative divergence *d*_2_ < *d*_1_. It thus follows that when the system has left a subspace with divergence *d*_1_ that it can never return to any subspace with divergence ≥ *d*_1_. It also follows that the network can only ever enter one of the many subspaces *S*_*i*_ with equal divergence *d*_*i*_ = *X* (Fig. 2F shows an example). Not all subspaces with lower *d*_*i*_ than the current subspace are reachable. This is because once a unit has become inactive by crossing its activation threshold, it will remain inactive. Together, this introduces a hierarchy of forbidden subspaces that the network traverses while in the exploration phase. Fig. 4A shows the hierarchy of the sets imposed by the network used in Fig. 2. This tree-like structure of subspaces constrains computation in such a way that at any point of time, only a limited number of choices can be made. As a consequence, once the network enters a certain forbidden subspace, a subset of other forbidden and permitted subspaces becomes unreachable (Fig. 4B). What those choices are depends on the input whereas the tree-like structure of the subspaces is given by the network connectivity.

**Figure 5:**
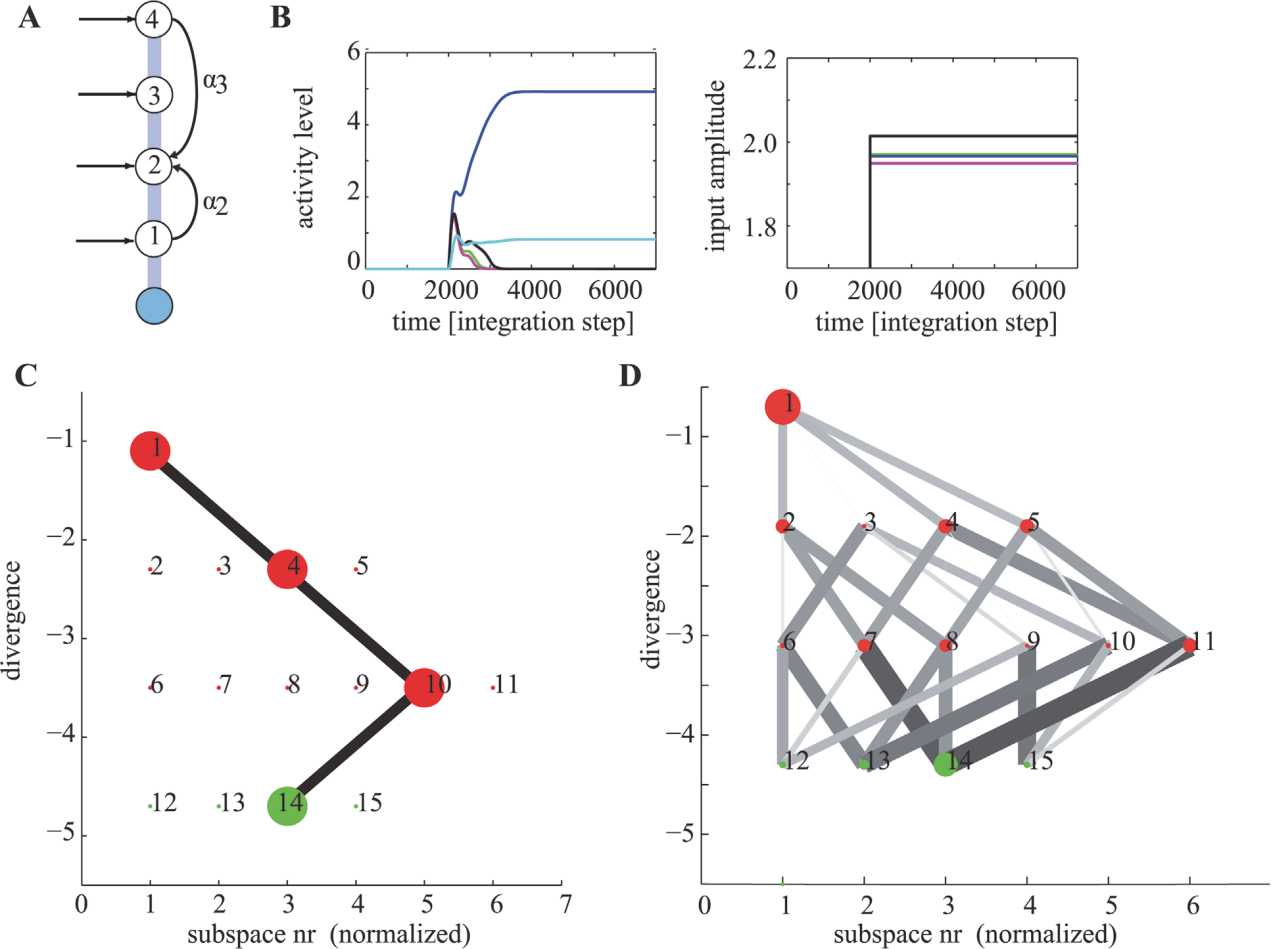
Steering of computation. (A) The same network as shown in Fig. 4A, with two additional excitatory connections *α*_2_ = 0.2 and *α*_3_ = 0.2. (B) Simulation of an individual run. Top shows the state and bottom the inputs to the network. Although the input is identical to Fig. 4B, the computation proceeds differently due to presence of the additional connections. (C) Trajectory through state space for the simulation shown in (C). The state space trajectory is initially identical to the simulation shown in (Fig. 4C), but then diverges. (D) All transitions made by the network. The transition probabilities are now biased and non-equal. Consequently, some permitted subspaces are reached more frequently than others.

Knowledge of how the network transitions through the hierarchy of forbidden subspaces can be used to systematically introduce biases into the computational process. Such additional steering of the computation can be achieved by adding connections in the network. Additional off-diagonal excitatory connections, for example, will make it more likely that a certain configuration is the eventual winner. An example of this effect is shown in Fig. 5, where adding two additional excitatory connections results in the network being more likely to arrive in a given permitted subspace than others. For identical inputs (compare Figs. 5B and 4B) the resulting permitted subspace can be different through such steering.

**Figure 6:**
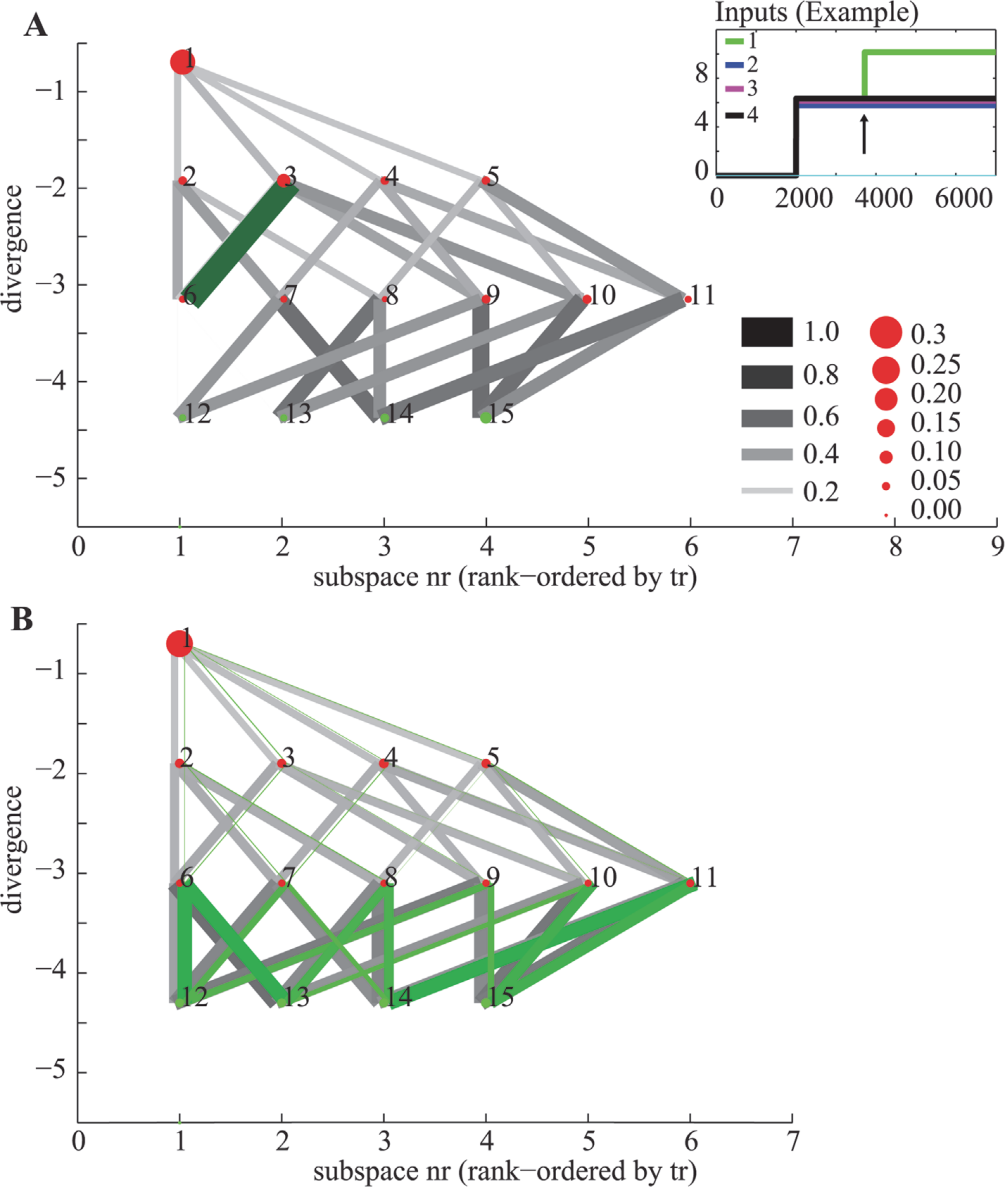
Steering of computation by external inputs. The network remains sensitive to changes in its input throughout the computation. Transitions are bi-directional: towards more negative and positive divergence are gray and green, respectively. (A) Example of a selective change in the input. Here, unit 4 receives additional external input only if the network enters forbidden subspace 6 (see inset). This change forces the network to make a transition to a subspace with less negative divergence (green). As a result, the eventual solution is more likely to be subspace 15. (B) Example of continuously changing inputs (input noise). Notice how noise introduces additional transitions towards less negative divergence at all stages. However, the general gradient towards more negative divergence persists: overall, 26% of transitions made the divergence more positive.

The network remains continuously sensitive to changes in the external input. This is important and can be used to steer the computation without changing the structure of the network. In the absence of changes in the external input, the network is unable to make transitions other than those which lead to subspaces with lower divergence. When the input changes, on the other hand, the network can make such changes. For example, if the inputs change as a consequence of the network entering a certain forbidden subspace, the network can selectively avoid making certain transitions (Fig. 6A). This will steer the computation such that some permitted subspaces are reached with higher likelihood. Noisy inputs similarly can lead to transitions which make divergence less negative. Neverthless, the large majority of transitions remains negative as long as noise levels are not too large. For example, repeating the same simulation but adding normally distributed noise with *σ* = 1 and *μ* = 0 resulted in 26% of transitions being against the gradient (see Fig. 6B for an illustration).

So far, we have illustrated how negative divergence and expansion jointly drive the computational process in a simple circuit of multiple excitatory neurons that have common inhibition. While this circuit alone is already capable of performing sophisticated computation,many computations require that several circuits interact with one another [23, 29] The concepts developed in this paper can also be applied to such compound circuits, because the circuit motifs and parameter bounds we describe guarantee that collections of these circuits will also possess forbidden and permitted subspaces and are thus also computing. The compound network is guaranteed to be dynamically bounded, which means that no neuron’s activity can escape towards infinity. This property of the collective system relies on two key aspects: i) collections of individual circuits with negative divergence also have negative divergence, and ii) collective stability [24, 30]. Together, these properties guarantee that collections of the motifs will compute automatically.

**Figure 7:**
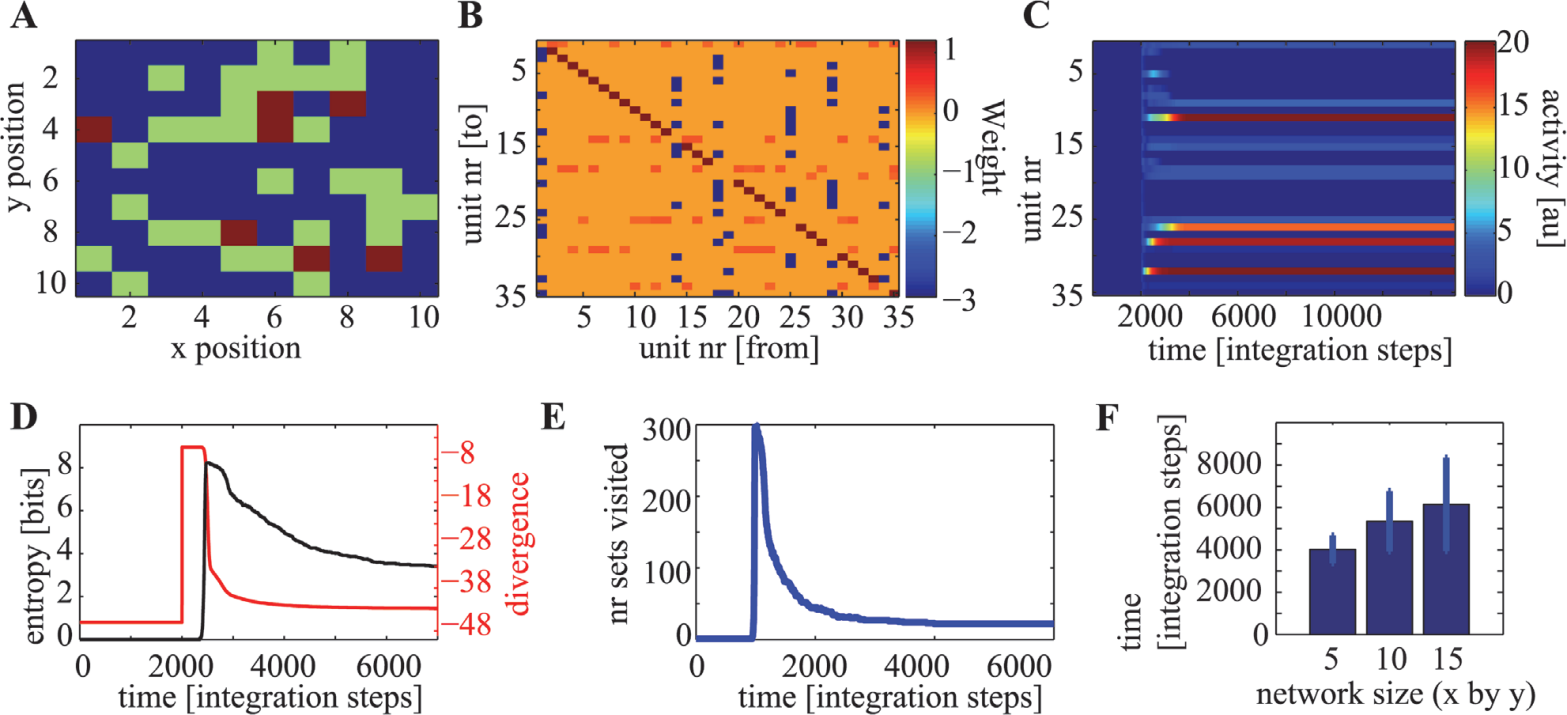
Large random networks spontaneously compute. (A) Each site in a 10×10 grid is either empty or occupied by an excitatory (green) or inhibitory (red) neurons (probabilistically). (B) Connectivity matrix. (C) Example run. (D) Entropy and divergence as a function of time for random initial conditions. (E) The number of different subspaces visited reduces as a function of time similarly to the entropy. (F) Time ±*s.d*. till the network first enters a permitted subspace as a function of network size, specified as grid with. 100 runs of randomly assembled networks were simulated for each size. The larger the network, the longer the network spent going through the forbidden subspaces. Panels A–E show an example of grid size 10. See Methods for simulation details.

Consider a network assembled by randomly placing instances of the two circuit motifs on a 2D plane and connecting them to each other probabilistically (Fig. 7A,B and methods). This random configuration results in some excitatory elements sharing inhibition via only one inhibitory motif, whereas others take part in many inhibitory feedback loops (Fig. 7B). This random circuit will compute spontaneously (Fig. 7C,D). It is not known a priori how many forbidden and permitted subspaces the network has, nor how many possible solutions it can reach. Nevertheless, it is guaranteed that the network will reduce entropy and eventually reach a permitted subspace (Fig. 7E). The more connections (constraints) that are added to the network the smaller the number of permitted subspaces, and generally the harder the computation will become. How long the computation will take to reach a permitted subspace depends on both the network size, and the number of connections (constraints). Generally, the smaller the number of permitted subspaces the harder the computation will be. The important point is that random instances of such circuits will always compute, which means they will always reach a permitted subspace (Fig. 7F).

## Discussion

The contribution of this paper has been to explore the fundamental role of instability in driving computation in networks of linear threshold units. Previous studies of computation in neural networks have focused on networks of sigmoidal units with symmetrical connectivity. Our networks of asymetrically connected LTNs draw attention to important features of computation that were not apparent in these previous models. The conditional selective behavior crucial for computation depends on the threshold nonlinearity of the LTN. However, in order to make use of these non-linearities the network must express substantial gain. Because the activation of LTNs is unbounded for positive inputs, the network can in principle produce very high activations through unstably high gain. In these networks, computation is expressed as passage through a sequence of unstable states. It is this dynamical trajectory by which the network computes [1, 2, 31]. Despite this essential instability, the system does not escape, but remains bounded in its behavior. In this paper we have analyzed why this is so. We find that the instabilities are self limiting, and that the overall process of computation is systematically quenched by Gaussian divergence. Contraction analysis provides explicit tools to quantify both instantaneous rates of exponential convergence to limiting states or trajectories, and divergence rates from specific subspaces. Here, we use these tools to analyze the unstable phase of the dynamics. This phase is crucial, because computation is inseparable from instability. Here we have made steps towards characterizing and explaining these phenomena.

The type of dynamical system we consider can implement soft-WTA type behavior, amongst others. This makes our framework applicable to the extensive body of literature on this type of network [32–38]. While simple, the soft-WTA is a powerful computational primitive that offers the same computational power than a multi-layer perceptron [32]. Key aspects of what makes WTA-networks powerful are high network gain, which allows computations that require sparsification, and also provides stability. While the computational power of WTAs has long been recognized and exploited to implement simple cognitive behaviors [23, 29, 39], it has remained unclear what it means to compute in such networks. Here, we provide such understanding in terms of a dynamical system. This system is physically realizable by realistic neurons and their connections. Other work in this direction has focused on abstract mathematic models [36, 40–43], and less on physically realizable dynamical computation. More recently, others [44–46] have offered useful models for understanding the principles whereby the brain may attain cognition, but these approaches do not offer methods for implementing such algorithms as physical computation in neuronal circuits.

The advances of this paper can be seen in contrast with classical assumptions concerning the form of activation functions, continuous sensitivity to input, and symmetry of connections. For example, the behavior of our LTN networks can be contrasted with networks of the kind originally proposed by Hopfield [20] that allow no self-connections (*w*_*ii*_ = 0, ∀*i*), have symmetric connectivity (*w*_*ij*_ = *w*_*ji*_), and their activation function is bounded on both sides. This guarantees bounded dynamics by construction, allowing such networks to express high gain by virtue of a steep activation function rather than through connections of the network. However, a consequence of this is that when it operates with high gain the network operates in saturation and thus becomes insensitive to input apart from initial conditions. Such networks have neither negative divergence nor rotational dynamics, which together with insensitivity to external input severely restricts their computational abilities as well as systematic design.

Importantly, our networks are continuously sensitive to their inputs. These external inputs are a combination of signal and noise and can transfer the network from one subspace to an other at any point of time and this transfer can be against the gradient imposed by negative divergence. Non-autonomous systems continuously interact with their environment, for which continuous sensitivity to input is crucial. Systems of asymmetrically interacting linear threshold units are well suited for this situation. This is because their non-saturating units make the system adaptive to the input amplitudes and sensitivity to inputs is conditional on the current state, i.e. only the inputs contributing to the dynamics of the currently active state influence the dynamics.

Although there has been a considerable amount of work on symmetric networks, biological neuronal networks are always asymmetric because of inhibitory neurons. Also, the inhibitory inputs to an excitatory neuron can be substantially stronger than the excitatory inputs. This results in particularly strong asymmetry, a property with many implications for computation in such networks [47]. The theoretical study of networks with defined cell types (excitatory or inhibitory) thus requires asymmetric connectivity. Previous studies have used infinitely fast all-to-all inhibition to circumvent this problem, which results in symmetric connectivity but lacks defined cell types. Such networks allow dynamically bounded activity for linear threshold units [16, 48]. Apart from being biologically unrealistic, such networks can only express limited gain and are thus computationally very limited [47]. By contrast, our networks express high gain and dynamic instabilities during the exploration phase. Their asymmetric connections provide the rotational dynamics that keep their activity bounded despite this high gain. It is worth noting that many powerful algorithms, such as e.g. the Kalman filter [49, 50] also rely on negative feedback and strongly asymmetric connectivity.

The dynamics of the exploration phase are highly structured because the different forbidden subspaces are systematically related to one another. Indeed, the subspaces are ordered in a hierarchy through which the dynamics proceed. At any point in this hierarchy only a limited and known set of subspaces can be entered next (unless the external input changes). The systematic understanding of the unstable dynamics driving exploration can be used to steer and modify the computational trajectory while it is in process, rather than only when a solution has been found. The network can influence its environment continuously as a function of the forbidden subspaces it traverses, for example by executing a specific action whenever a particular subspace is entered. This feature can be used to make the computations of several networks dependent on each other. For example. to enforce dependencies between several ongoing computations such as, “all solutions must be different”.

The connections of the network are the constraints imposed on the computation. The more connections per neuron, the fewer possible solutions exist and the harder (slower) the computation is. From this point of view, the networks we describe perform constraint satisfaction, which is a hard computational problem and which has been proposed as an abstract model of computation [51, 52]. Connections can be inserted systematically to forward program specific algorithms and behaviors [23, 24, 29], randomly or a combination thereof. Either way, the system will compute [53], but in the former case will execute specific algorithms while in the later the algorithm is unknown.

The constraints active at any point of time depend on the state of the network as expressed by the effective connectivity of the network expressed by the switching matrix. Every time the network changes state, the switching matrix changes. Dynamically, the same concept can be applied: the effective Jacobian jointly expresses all the currently activity constraints for a given state. Only if the possible state(s) of a network are known is it possible to determine the effective Jacobian. An important implication is that to understand the underlying algorithm that drives the computation performed by a group of neurons knowledge of the structural connectivity is not sufficient [54–56]. This is because connectivity alone does not determine the possible states of the network.

The circuit motifs and parameter bounds we describe guarantee that collections of these circuits will also possess forbidden and permitted subspaces and thus are computing. By collections we mean multiple copies of the same motifs that are in addition connected to each other, as for example in the random network discussed in the results. This is important because collections of these motifs will compute automatically, a property we refer to as collective computation. This makes it practical to design large-scale computing systems without having to perform global analysis to guarantee both the type of instability required for computation as well as stability of the solutions.

It is important to note that one need not commit to a particular circuit motif beyond guaranteeing that both forbidden and permitted subspaces exist in the way we define them. While a network composed of such motifs but otherwise connected randomly will always compute, the individual states do not have meaning nor is the algorithm that the network computes known. However, the states that the network proceeds through while it computes are systematically related to each other. Consequently, assigning a meaningful interpretation to a few key states will make all states meaningful. A similar approach is used in reservoir computing, where states are first created and only later assigned with meaning by learning mechanisms [57]. A key next step will be to discover how linking specific forbidden subspaces with motor actions that in turn change the input to the system allow a computation to remain continuously sensitive to the environment while it proceeds. An other next step is to discover how several interacting systems can bias each others computations systematically to reach a solution that is agreeable to all while satisfying the local constraints of each computation.

The unstable positive feedback and cross inhibition that underly expansion and divergence are common motifs found in many molecular, cellular and neuronal networks [58]. Therefore all such systems follow the very simple organizational constraints that combine these motifs. This will lead such circuits to compute spontaneously and thereby to reduce their state entropy as is characteristic of living systems.

## Methods

### Numerical methods

All simulations were performed with Euler integration with δ = 0.01, thus *τ* is equivalent to 100 integration steps. All times in the figures and text refer to numerical integration steps. Unless noted otherwise, the external inputs *I*_*i*_(*t*) were set to 0 at *t* = 0 and then to a constant non-zero value at *t* = 2000. The constant value *I*_*i*_ was drawn randomly and i.i.d. from N(6,1σ). All simulations were implemented in MATLAB.

In cases where noise was added, the noise was supplied as an additional external input term N(0,σ) with *σ* = 1.

$$\tau {\dot{x}}_{i}+G_{i}x_{i}=f\left(I_{i}+N\left(0,\sigma \right)+\alpha_{1}x_{i}+\alpha_{2}x_{i-1}+\alpha_{2}x_{i+1}-\beta_{1}x_{N}\right)$$ (15)

A new noise sample is drawn from the random variable every *τ* to avoid numerical integration problems.

#### Large network simulation

A 2D square of dimension 10 by 10 spots is created. Each spot is occupied by a neuron with *P* = 0.4. If a spot is occupied, it is excitatory with *P* = 0.8 and inhibitiory otherwise. Connectivity between units is generated with the following constraints: All excitatory units receive excitation from themselfs with *α*_1_. Further, each excitatory unit receives inhibiton from and excites up to 8 randomly chosen inhibitory neurons with probability *P* = 0.4. It is very unlikly that an excitatory unit is not connected to any inhibitory neuron at all (*P* = (0.6)^8^).

### Entropy

The information entropy *H*(*t*) is

$$H\left(t\right)=-\sum_{i}p_{i}\left(t\right)log_{2}\left(p_{i}\left(t\right)\right)$$ (16)

The sum is over all subspaces *i* and *p*_*i*_(*t*) is the probability of the network being in subspace *i* at time *t*, over random initial conditions (we used 1000 runs). By definition, we take 0*log*_2_(0) = 0.

### Analytical methods

The principal analytical tool used is contraction analysis [17, 59–61]. In this section, we briefly summarize the application of contraction analysis to analyzing asymmetric dynamically bounded networks [24]. Essentially, a nonlinear time-varying dynamic system will be called *contracting* if arbitrary initial conditions or temporary disturbances are forgotten exponentially fast, i.e., if trajectories of the perturbed system return to their unperturbed behavior with an exponential convergence rate. Relatively simple algebraic conditions can be given for this stability-like property to be verified, and this property is preserved through basic system combinations and aggregations.

A nonlinear contracting system has the following properties [17, 59–61]

- global exponential convergence and stability are guaranteed
- convergence rates can be explicitly computed as eigenvalues of well-defined Hermitian matrices
- combinations and aggregations of contracting systems are also contracting
- robustness to variations in dynamics can be easily quantified

Consider now a general dynamical system in ℝ^*n*^,

$$\dot{x}=f\left(x,t\right)$$ (17)

with **f** a smooth non-linear function. The central result of Contraction Analysis, derived in [17] in both real and complex forms, can be stated as:

**Theorem** Denote by $\frac{\partial \text{f}}{\partial \text{x}}$ the Jacobian matrix of **f** with respect to **x**. Assume that there exists a complex square matrix **Θ**(*x*, *t*) such that the Hermitian matrix **Θ**(*x*, *t*)^*T^ **Θ**(*x*, *t*) is uniformly positive definite, and the Hermitian part **F**_*H*_ of the matrix

$$F=\left(\dot{\Theta }+\Theta \frac{\partial \text{f}}{\partial \text{x}}\right)\Theta^{-1}$$

is uniformly negative definite. Then, all system trajectories converge exponentially to a single trajectory, with convergence rate |sup_**x**, *t*_λ_max_(**F**_*H*_)| > 0. The system is said to be *contracting*, **F** is called its *generalized Jacobian*, and **Θ**(*x*, *t*)^*T^ **Θ**(*x*, *t*) its contraction *metric*. The contraction rate is the absolute value of the largest eigenvalue (closest to zero, although still negative) *λ* = ∣*λ*_*max*_(**F**_*H*_)∣.

In the linear time-invariant case, a system is globally contracting if and only if it is strictly stable, and **F** can be chosen as a normal Jordan form of the system, with **Θ** a real matrix defining the coordinate transformation to that form [17]. Alternatively, if the system is diagonalizable, **F** can be chosen as the diagonal form of the system, with **Θ** a complex matrix diagonalizing the system. In that case, **F**_*H*_ is a diagonal matrix composed of the real parts of the eigenvalues of the original system matrix. Here, we choose Θ = **Q** ^−1^ where **Q** is defined based on the eigendecomposition **J** = **QΛQ** ^−1^.

The methods of Contraction Analysis were crucial for our study for the following reasons: i) Contraction and divergence rates are exponential guarantees rather than asymptotic (note that the more familiar Lyapunov exponents can be viewed as the average over infinite time of the instantaneous contraction rates in an identity metric). ii) No energy function is required. Instead, the analysis depends on a metric **Θ** that can be identified for a large class of networks using the approach outlined. iii) The analysis is applicable to non-autonomous systems with constantly changing inputs.

The boundary conditions for a WTA-type network to be contracting as well as to move exponentially away from non-permitted configurations were derived in detail in [24]. They are:

$$\begin{array}{c} 1<\alpha <2\sqrt{\beta_{1}\beta_{2}}\\ \frac{1}{4}<\beta_{1}\beta_{2}<1 \end{array}$$ (18)

#### Forbidden subspaces have mixed eigenvalues

When the system resides in a forbidden subspace, the maximal eigenvalue of the effective Jacobian is positive and its divergence is negative. A forbidden subspace thus has a mixed eigenvector **v** associated with the maximal eigenvalue *λ*_*max*_. A mixed eigenvector is one where at least one entry is strictly negative and at least one strictly positive.

Proof by contradiction: Assume all components *v*_*i*_ ≥ 0. Consider a constant external input where the system starts along one of the eigenvectors **v**. In this case, the state of the network is determined entirely by this eigenvector and would grow towards infinity without ever changing dimensionality. However, the conditions for a forbidden subspace do not permit this because **Vx** = 0 ensures that the system escapes this subspace exponentially fast (see Eqs 7–8). Thus in a forbidden subspace, by definition, the state cannot grow towards infinity and the eigenvector **v** must be mixed.

#### Rotation versus expansion—Example

This example illustrates the role of rotation.

$$\left[\begin{array}{cc} 1 & a\\ -a & -2 \end{array}\right]=\left[\begin{array}{cc} 1 & 0\\ 0 & -2 \end{array}\right]+a\left[\begin{array}{cc} 0 & 1\\ -1 & 0 \end{array}\right]$$ (19)

In the above, divergence is −1. Whether the system is linearly stable depends on the value of a. If the determinant −2 + *a* ^2^ > 0, this system is stable. Thus the system is stable if *a* ^2^ > 2, i.e. if the rotation is “fast enough”.
